# Clinical outcomes of patients requiring ventilatory support in Brazilian intensive care units: a multicenter, prospective, cohort study

**DOI:** 10.1186/cc12594

**Published:** 2013-04-04

**Authors:** Luciano CP Azevedo, Marcelo Park, Jorge IF Salluh, Alvaro Rea-Neto, Vicente C Souza-Dantas, Pedro Varaschin, Mirella C Oliveira, Paulo Fernando GMM Tierno, Felipe dal-Pizzol, Ulysses VA Silva, Marcos Knibel, Antonio P Nassar, Rossine A Alves, Juliana C Ferreira, Cassiano Teixeira, Valeria Rezende, Amadeu Martinez, Paula M Luciano, Guilherme Schettino, Marcio Soares

**Affiliations:** 1 Research and Education Institute, Hospital Sírio-Libanês, Rua Cel. Nicolau dos Santos 69 - São Paulo, 01308-060, Brazil; 2 ICU, Emergency Medicine Department, Hospital das Clinicas da Faculdade de Medicina da Universidade de São Paulo, Av. Eneas Carvalho Aguiar 255, São Paulo, 05403-000, Brazil; 3 D'Or Institute for Research and Education, Rua Dinis Cordeiro 30, Rio de Janeiro, 22281-100, Brazil; 4 CEPETI - Centro de Estudos e Pesquisas em Terapia Intensiva, Rua Monte Castelo 366, Curitiba, 82530-200, Brazil; 5 ICU, Instituto Nacional de Câncer - Hospital do Câncer I, Praça Cruz Vermelha 23, Rio de Janeiro, 20230-130, Brazil; 6 ICU, Hospital Pasteur, Av. Amaro Cavalcanti 495, Rio de Janeiro, 20735-040, Brazil; 7 ICU, Surgical Emergency Department, Hospital das Clinicas da Faculdade de Medicina da Universidade de São Paulo, Av. Eneas Carvalho Aguiar 255, São Paulo, 05403-000, Brazil; 8 ICU, Hospital São José, Rua Cel. Pedro Benedet 630, Criciúma, 88801-250, Brazil; 9 ICU, Fundação Pio XII, Hospital de Câncer de Barretos, Rua Antenor Duarte Vilela 1331, Barretos, 14780-000, Brazil; 10 ICU, Hospital São Lucas, Travessa Frederico Pamplona 32, Rio de Janeiro, 22061-080, Brazil; 11 ICU, Hospital São Camilo Pompéia, Av. Pompeia 1178, São Paulo, 05024-000, Brazil; 12 ICU, Hospital Regional Público do Araguaia - Av. Brasil, s/n Qd. 30, Redenção, 68552-735, Brazil; 13 ICU, Hospital A. C. Camargo, R. Professor Antônio Prudente 109, São Paulo, 01509-010, Brazil; 14 ICU, Hospital Moinhos de Vento, Rua Ramiro Barcelos 910, Porto Alegre, 90035-001, Brazil; 15 ICU, Hospital Geral de Roraima, Av. Brg. Eduardo Gomes 3388, Boa Vista, 69305-284, Brazil; 16 ICU, Hospital Espanhol, Av. Sete de Setembro 4161, Salvador, 40140-110, Brazil; 17 ICU, Hospital Estadual Américo Brasiliense, Alameda Dr. Aldo Lupo 1260, Américo Brasiliense, 14802-520, Brazil

## Abstract

**Introduction:**

Contemporary information on mechanical ventilation (MV) use in emerging countries is limited. Moreover, most epidemiological studies on ventilatory support were carried out before significant developments, such as lung protective ventilation or broader application of non-invasive ventilation (NIV). We aimed to evaluate the clinical characteristics, outcomes and risk factors for hospital mortality and failure of NIV in patients requiring ventilatory support in Brazilian intensive care units (ICU).

**Methods:**

In a multicenter, prospective, cohort study, a total of 773 adult patients admitted to 45 ICUs over a two-month period requiring invasive ventilation or NIV for more than 24 hours were evaluated. Causes of ventilatory support, prior chronic health status and physiological data were assessed. Multivariate analysis was used to identifiy variables associated with hospital mortality and NIV failure.

**Results:**

Invasive MV and NIV were used as initial ventilatory support in 622 (80%) and 151 (20%) patients. Failure with subsequent intubation occurred in 54% of NIV patients. The main reasons for ventilatory support were pneumonia (27%), neurologic disorders (19%) and non-pulmonary sepsis (12%). ICU and hospital mortality rates were 34% and 42%. Using the Berlin definition, acute respiratory distress syndrome (ARDS) was diagnosed in 31% of the patients with a hospital mortality of 52%. In the multivariate analysis, age (odds ratio (OR), 1.03; 95% confidence interval (CI), 1.01 to 1.03), comorbidities (OR, 2.30; 95% CI, 1.28 to 3.17), associated organ failures (OR, 1.12; 95% CI, 1.05 to 1.20), moderate (OR, 1.92; 95% CI, 1.10 to 3.35) to severe ARDS (OR, 2.12; 95% CI, 1.01 to 4.41), cumulative fluid balance over the first 72 h of ICU (OR, 2.44; 95% CI, 1.39 to 4.28), higher lactate (OR, 1.78; 95% CI, 1.27 to 2.50), invasive MV (OR, 2.67; 95% CI, 1.32 to 5.39) and NIV failure (OR, 3.95; 95% CI, 1.74 to 8.99) were independently associated with hospital mortality. The predictors of NIV failure were the severity of associated organ dysfunctions (OR, 1.20; 95% CI, 1.05 to 1.34), ARDS (OR, 2.31; 95% CI, 1.10 to 4.82) and positive fluid balance (OR, 2.09; 95% CI, 1.02 to 4.30).

**Conclusions:**

Current mortality of ventilated patients in Brazil is elevated. Implementation of judicious fluid therapy and a watchful use and monitoring of NIV patients are potential targets to improve outcomes in this setting.

**Trial registration:**

ClinicalTrials.gov NCT01268410.

## Introduction

Acute respiratory failure is frequent and commonly a severe organ dysfunction occurring in the intensive care unit (ICU) [1]. Under this circumstance, invasive or non-invasive mechanical ventilation (MV) are life-sustaining interventions [2]. However, despite significant advances in ventilatory support [3], it remains associated with elevated mortality [4] and a significant impairment in the patients' quality of life in the post-ICU setting [5]. Therefore, information about the epidemiological aspects of patients under MV is important from both clinical and health policy perspectives. However, most studies on the epidemiology of ventilatory support are outdated or were carried out before significant developments in the field, such as lung protective ventilation [6] or the widespread application of non-invasive mechanical ventilation (NIV) [7-9]. Moreover, these studies were usually carried out in high-income countries and very few contemporary data from emerging countries are available [10-12]. Specifically from Brazil, a previous trial evaluated the mortality of patients with acute respiratory failure. However, this study was a single center trial carried out only in a tertiary hospital and just included individuals with invasive mechanical ventilation and not patients under NIV [11]. Comprehensive information about the clinical characteristics, outcomes and risk factors for mortality of these patients is essential to assist clinicians in the decision-making process and to allow better resource allocation. Therefore, we carried out a multicenter, observational cohort study in Brazilian ICUs to describe the clinical outcomes of patients submitted to ventilatory support as well as to identify variables associated with hospital mortality.

## Materials and methods

### Design and setting

The **E**pidemiology of **R**espiratory **I**nsufficiency in **C**ritical **C**are (ERICC) study was a multicenter prospective cohort study conducted in 45 Brazilian ICUs between 1 June 2011 and 31 July 2011. The study was coordinated by the Research and Education Institute from Hospital Sírio-Libanês, São Paulo and D'Or Institute for Research and Education, Rio de Janeiro. Invitations were sent to ICUs registered at the Brazilian Research in Intensive Care Network (BRICNet) database and 45 ICUs from 12 Brazilian states agreed to participate. Investigators and centers are listed at the acknowledgements section. The study was strictly observational and decisions related to patients' care were at the discretion of the attending ICU team. The study was approved by the institutional review board (IRB) at the coordinating center (Comitê de Ética em Pesquisa - CEPesq - approval number HSL 2010/51) and, subsequently, by the National Ethics Committee and local review boards at each participating site. The need for informed consent was waived or requested in some sites according to the local IRBs directives.

### Selection of participants and definitions

Patients aged ≥ 18 years old requiring ventilatory support for > 24 h during the first 48 h of ICU admission at the participating ICUs were included in the study. In the subgroup of patients undergoing NIV, only those that used this modality for at least 6 h/day were included. Patients with a previous tracheostomy, admitted for routine uncomplicated postoperative care (ICU stay < 48 h), readmissions and those with terminal conditions were not considered.

Demographic, clinical and laboratory data were collected during the ICU stay, including the main diagnosis for ICU admission, the reasons for and modality of ventilatory support (conventional MV or NIV), chronic health status, the Charlson Comorbidity Index [13], the need for vasopressors, dialysis, tracheostomy, the Simplified Acute Physiology Score 3 (SAPS 3) [14] and the Sequential Organ Failure Assessment (SOFA) score [15]. Patients who first received NIV, irrespective of its duration, and subsequently required endotracheal intubation were considered as NIV failure. The cumulative fluid balance over the first 72 h of ICU stay was also calculated. Sepsis was diagnosed using the current definitions [16]. The patient was considered to have an infection when there were clinical, laboratory, radiological and microbiological findings suggesting the presence of infection that justified the administration of antibiotics (excluding prophylaxis) [17]. Acute respiratory distress syndrome (ARDS) was defined and classified according to the Berlin definition [18]. The main outcome of interest in the current study was all cause in-hospital mortality.

### Data entry and processing

Data were collected using a web-based specific and standardized electronic case report form. Each investigator and research coordinator was provided access to the website, where all study documentation, including a comprehensive manual describing data collection requirements and variable definitions, was available. A central office was accessible through telephone and email contact to provide support to investigators. Local investigators were responsible for training local staff for data collection, supervising data collection, controlling for data completeness and quality.

Data consistency was assessed through a rechecking procedure of a 5% random sample of patients. Data were screened in detail by three investigators (LCA, MS, MP) for missing information, implausible and outlying values, logical errors and insufficient details. In case of unconformity, local investigators were contacted to provide the requested information.

### Statistical analysis

Standard descriptive statistics were used to describe the study population. Continuous variables were reported as median (25% to 75% interquartile range, IQR). Univariate and multivariate analysis using a binary logistic regression were used to identify factors associated with the dependent variables (hospital mortality or NIV failure) [19]. We also carried out analysis of SOFA score excluding the respiratory component to reduce interaction with ARDS in the multivariate analysis and to assess the severity of associated organ failures. Linearity between each continuous variable and the dependent variable was demonstrated using locally weighted scatterplot smoothing (LOWESS) [19]. In case of nonlinearity, the variable was transformed or stratified according to the analysis of the functional form and clinical significance. For categorical variables with multiple levels, the reference level was attributed to the one with the lowest probability of the dependent variable. Variables yielding *P-*values < 0.2 by univariate analysis were entered in the multivariate analysis to estimate the independent association of each covariate with the dependent variable. Results were summarized as odds ratios (OR) and respective 95% confidence intervals (CI). Possible interactions were tested. Two-tailed *P-*values < 0.05 were considered statistically significant.

## Results

### Characteristics of study population

**Figure 1:**
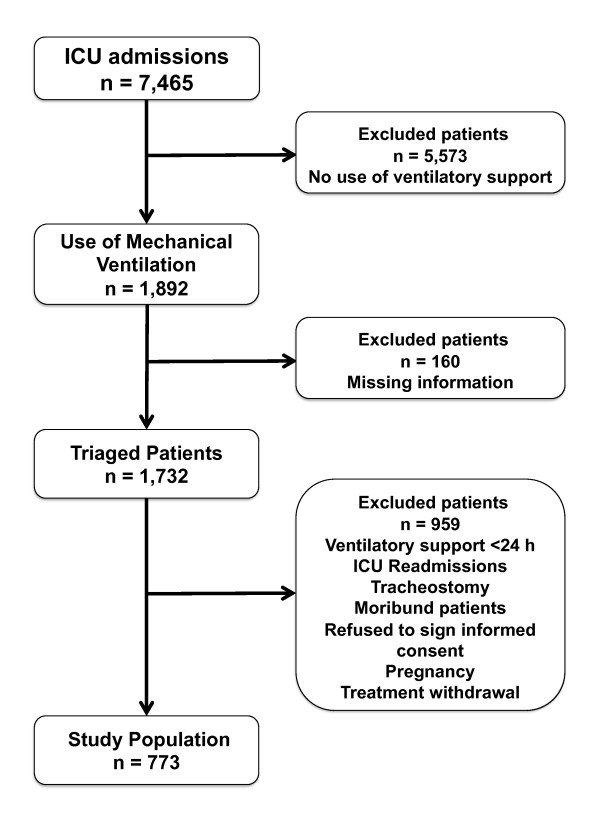
**Flowchart of the study**.

The study flowchart is shown in Figure 1. A total of 773 patients fulfilled the eligibility criteria of the study and were therefore evaluated. Their main characteristics are depicted in Table 1. The most frequent diagnoses at ICU admission were pneumonia (27%), neurological diseases (19%), non-pulmonary sepsis (12%) and obstructive pulmonary disease (6%). Patients were admitted to the ICU at a median of one (zero to three) day after hospital admission. Median SAPS 3 score was 62 (52 to 72) points and the probability of death estimated by the global equation was 40 ± 24%. Using the customized equation for countries from Central and South America, the probability of death estimated by SAPS 3 was 52 ± 26%. Most patients (67%) used vasopressors during their stay in the ICU and 19% required renal replacement therapy (RRT).

**Table 1:** Patients' characteristics and univariate analysis of factors associated with hospital mortality

Characteristic	All patients (*n *= 773)	Survivors (*n *= 451)	Non-survivors (*n *= 322)	OR (CI 95%)	*P*-value
**General**					
Age - yo	62 (43 to 76)	58 (35 to 72)	69 (53 to 80)	1.03 (1.02 to 1.04)	< 0.001
Male gender - n (%)	435 (56)	260 (58)	175 (54)	0.87 (0.66 to 1.17)	0.362
Ideal weight - Kg	61 (52 to 69)	62 (54 to 71)	61 (52 to 67)	0.98 (0.97 to 0.99)	0.034
Admission SAPS 3 score (points)	62 (52 to 72)	58 (49 to 67)	68 (57 to 77)	1.04 (1.03 to 1.06)	< 0.001
SOFA score on Day 1 (points)	8 (5 to 10)	7 (4 to 9)	8 (7 to 11)	1.17 (1.12 to 1.23)	< 0.001
SOFA score excluding respiratory component (points)	6 (4 to 8)	5 (3 to 8)	7 (5 to 9)	1.18 (1.13 to 1.24)	< 0.001
Charlson Comorbidity Index (points)					
0	314 (41)	227 (50)	87 (27)	Ref.	
1 to 2	288 (37)	160 (36)	128 (40)	0.23 (0.15 to 0.34)	< 0.001
> 2	171 (22)	64 (14)	107 (33)	0.48 (0.33 to 0.70)	
LOS prior ICU - days	1 (0 to 3)	1 (0 to 2)	1 (0 to 4)	1.03 (1.01 to 1.05)	0.009
					
**Admission source**					
Ward - n (%)	242 (31)	119 (26)	122 (38)	Ref.	
Emergency room - n (%)	367 (47)	225 (50)	142 (44)	0.62 (0.45 to 0.86)	0.004
Operation room - n (%)	164 (22)	107 (24)	58 (18)	0.54 (0.36 to 0.81)	0.003
					
**Admission Diagnosis**					
Pneumonia - n (%)	207 (27)	111 (25)	96 (30)	0.63 (0.32 to 1.23)	0.177
Neurological - n (%)	146 (19)	73 (16)	73 (23)	0.70 (0.41 to 1.22)	0.210
Non-pulmonary sepsis - n (%)	90 (12)	41 (9)	49 (15)	0.77 (0.49 to 1.19)	0.238
Asthma/COPD - n (%)	50 (6)	33 (7)	17 (5)	Ref.	
Cardiogenic pulmonary edema - n (%)	43 (6)	24 (5)	19 (6)	0.86 (0.42 to 1.75)	0.681
Extracranial trauma - n (%)	41 (5)	24 (5)	17 (5)	1.22 (0.76 to 1.95)	0.415
Hypovolemic/cardiogenic shock - n (%)	37 (5)	23 (5)	14 (4)	0.64 (0.27 to 1.55)	0.326
Aspirative syndromes - n (%)	26 (3)	17 (4)	9 (4)	0.96 (0.48 to 1.92)	0.915
Others - n (%)	133 (17)	73 (16)	60 (18)	0.74 (0.34 to 1.56)	0.431
					
**Comorbidities**					
Hypertension - n (%)	328 (42)	167 (37)	161 (50)	1.71 (1.28 to 2.29)	< 0.001
Diabetes - n (%)	177 (23)	81 (18)	96 (30)	1.95 (1.39 to 2.73)	< 0.001
Heart failure - n (%)	67 (9)	27 (6)	40 (12)	2.23 (1.34 to 3.73)	0.002
Chronic renal failure - n (%)	51 (7)	16 (4)	35 (11)	3.32 (1.80 to 6.10)	< 0.001
Chronic renal failure in dialysis n (%)	15 (2)	7 (2)	8 (2)	1.62 (0.58 to 4.50)	0.359
Neoplasm - n (%)	162 (21)	66 (15)	96 (30)	1.91 (1.38 to 2.65)	< 0.001
AIDS - n (%)	13 (2)	7 (2)	6 (2)	1.21 (0.40 to 3.63)	0.736
COPD - n (%)	80 (10)	47 (10)	33 (10)	0.98 (0.61 to 1.57)	0.938
					
**Ventilatory support category**					
Successful NIV - n (%)	70 (9)	57 (13)	13 (4)	Ref.	
NIV failure - n (%)	81 (10)	40 (8)	41 (13)	4.49 (2.14 to 9.45)	< 0.001
Invasive MV - n (%)	622 (80)	354 (78)	268 (82)	3.32 (1.78 to 6.19)	< 0.001
					
**Respiratory Data at first day**					
Pressure controlled mode - n (%)	371 (48)	208 (34)	163 (27)	1.09 (0.79 to 1.51)	0.603
Volume controlled mode - n (%)	186 (24)	104 (17)	82 (13)	1.06 (0.75 to 1.49)	0.742
Pressure support mode - n (%)	54 (7)	34 (6)	20 (3)	0.76 (0.42 to 1.35)	0.349
Tidal volume (ideal weight) - mL/kg	7.5 (6.1 to 8.7)	7.3 (6.0 to 8.7)	7.5 (6.6 to 8.9)	1.00 (0.90 to 1.11)	0.992
Plateau pressure - cmH_2_O	20 (17 to 24)	20 (17 to 24)	20 (16 to 24)	0.99 (0.95 to 1.04)	0.746
PEEP - cmH_2_O	6 (5 to 8)	6 (5,8)	6 (5 to 8)	0.95 (0.87 to 1.03)	0.206
PaO_2_/FiO_2 _ratio	257 (185 to 330)	260 (196 to 346)	250 (167 to 316)	0.99 (0.99 to 1.00)	0.014
ARDS - n (%)	242 (31)	116 (26)	126 (39)	1.50 (1.06 to 2.12)	0.021
Length of ventilatory support - days	5 (3 to 10)	5 (2 to 8)	6 (3 to 12)	1.02 (1.01 to 1.04)	0.005
					
**Support during ICU stay**					
RRT - n (%)	148 (19)	47 (10)	101 (31)	3.91 (2.67 to 5.75)	< 0.001
Vasopressors - n (%)	521 (67)	265 (60)	256 (79)	2.83 (2.02 to 3.98)	< 0.001
Cumulative fluid balance (72 h) - L	2.9 (0.8 to 5.4)	2.6 (0.8 to 4.6)	3.7 (1.1 to 6.2)	1.05 (1.01 to 1.10)	0.011
					
**Outcomes**					
ICU mortality - n (%)	260 (34)	-----------	-----------	-----------	----------
Hospital mortality - n (%)	322 (42)	-----------	-----------	-----------	----------
ICU LOS - days	10 (6 to 18)	10 (6 to 17)	10 (5 to 19)	1.01 (1.00 to 1.02)	0.047
Hospital LOS - days	20 (11 to 34)	23 (14 to 36)	16 (7 to 30)	0.99 (0.98 to 0.99)	0.009

Abbreviations: AIDS, Acquired Immunodeficiency Syndrome; ARDS, Acute Respiratory Distress Syndrome; COPD, Chronic Obstructive Pulmonary Disease; ICU, Intensive care unit; LOS, Length-of-stay; MV, Mechanical ventilation; NIV, Non-invasive ventilation; PEEP, Positive End-Expiratory Pressure; RRT, Renal Replacement Therapy; SAPS, Simplified Acute Physiology Score; SOFA, Sequential Organ Failure Assessment.

### Ventilatory support

**Figure 2:**
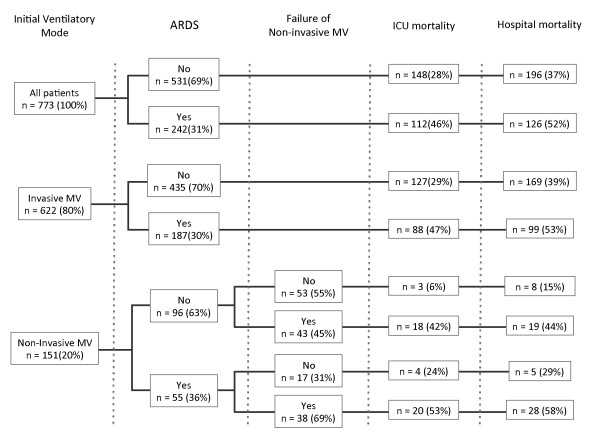
**ICU and hospital mortality rates according to ventilatory support, ARDS diagnosis and NIV failure**. ARDS, acute respiratory distress syndrome; ICU, intensive care unit; MV, mechanical ventilation.

Invasive MV was initially used in 80% (*n *= 622) of the patients and NIV was used in the remaining 20% (*n *= 151) of the patients as the initial ventilatory support (Table 1 and Figure 2). Of the later, 81 (54%) patients failed NIV support and were subsequently intubated for invasive MV. Ventilatory modes used initially in patients who received invasive MV were pressure-controlled ventilation (*n *= 371, 60%), volume-controlled ventilation (*n *= 186, 30%), pressure-support ventilation (*n *= 54, 9%) and others (*n *= 11, 1%). Median tidal volume was 7.5 (6.1 to 8.7) mL/kg of predicted body weight and plateau pressures were below 30 cmH_2_O in the vast majority of the patients.

### Outcome analysis

The overall ICU and hospital mortality rates were 34% and 42%, respectively (Figure 2 and Table 1). In the univariate analysis, age, ideal body weight, SOFA score at day 1, SAPS 3 score, Charlson comorbidity index, hospital length of stay before ICU, admission from the emergency room and from the operating room were associated with hospital mortality. Additionally, NIV failure, lower PaO_2_/FiO_2 _ratio, ARDS diagnosis, tracheostomy, duration of ventilatory support, need for vasopressors and renal replacement therapy (RRT), cumulative fluid balance and maximal blood lactate concentrations were also associated with hospital mortality (Table 1). In multivariate analysis, older age, higher SOFA scores (without respiratory component at Day 1), Charlson comorbidity index > 2, moderate to severe ARDS, NIV failure, use of invasive MV, higher lactate concentrations and both very negative or positive cumulative fluid balance over the first 72 hours of ICU stay were independently associated with increased hospital mortality (Table 2).

**Table 2:** Factors associated with hospital mortality in a multivariate analysis

Parameter	OR	CI 95%	*P*-value
Age	1.03	1.01 to 1.03	< 0.001
SOFA Score excluding respiratory component	1.12	1.05 to 1.19	< 0.001
			
Charlson comorbidity index			0.002
0	ref	ref	
1 to 2	1.37	0.92 to 2.04	0.124
> 2	2.30	1.28 to 3.17	< 0.001
			
ARDS			0.037
Absence ARDS	ref	ref	
ARDS mild	1.09	0.68 to 1.73	0.721
ARDS moderate	1.92	1.11 to 3.35	0.02
ARDS severe	2.12	1.02 to 4.41	0.045
			
Mechanical ventilation			0.004
Successful NIV	ref	ref	
NIV failure	3.96	1.74 to 8.99	0.001
Invasive MV	2.67	1.32 to 5.39	0.006
			
Cumulative fluid balance (72 h)			0.005
< -1.5 L	3.08	1.47 to 6.48	0.003
-1.5 L to +1.5 L	ref	ref	
> +1.5 L to +5.0 L	1.84	1.11 to 3.04	0.018
> 5.0 L	2.44	1.39 to 4.28	0.002
			
Lactate levels (Ln + 0.5)	1.78	1.27 to 2.51	0.001

Abbreviations: ARDS, Acute Respiratory Distress Syndrome; MV, Mechanical ventilation; NIV, Non-invasive ventilation; SOFA, Sequential Organ Failure Assessment Score. Area under receiver operating curve for predicted mortality (95% CI): 0.77 (0.73 to 0.81), *P *< 0.001. Hosmer-Lemeshow Χ^2^: 8.535, *P *= 0.383.

### ARDS diagnosis according to the Berlin definition

**Figure 3:**
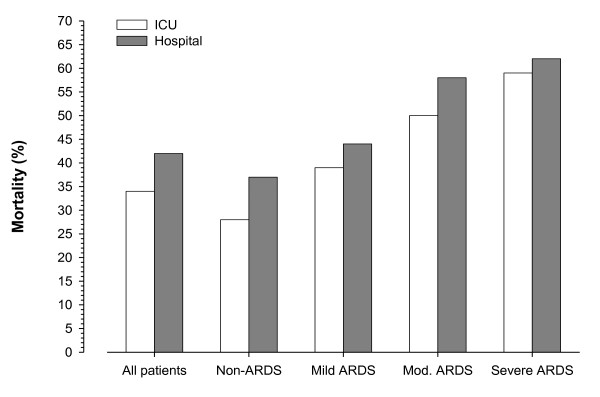
**ICU and hospital mortality rates according to the Berlin definition of ARDS**. *P *< 0.001 (Pearson Chi-square test) for the comparison of hospital mortality and ARDS classification. ARDS, acute respiratory distress syndrome; ICU, intensive care unit.

ARDS was diagnosed in 242 (31%) patients (Figure 2). Of these, 77% were supported with invasive MV and 23% received NIV as the initial ventilatory support. The rate of NIV failure in ARDS patients was 69%, as compared to 45% in non-ARDS patients (*P *= 0.007). ICU and hospital mortality in the ARDS population was 46% and 52%, respectively (Figure 2). In Figure 3, we depicted the ICU and hospital mortality rates for each category of ARDS. The combined ICU mortality for ARDS moderate and severe (the former definition of ARDS [20]) was 55% and the hospital mortality was 60%.

### Non-invasive ventilation characteristics and failure

**Figure 4:**
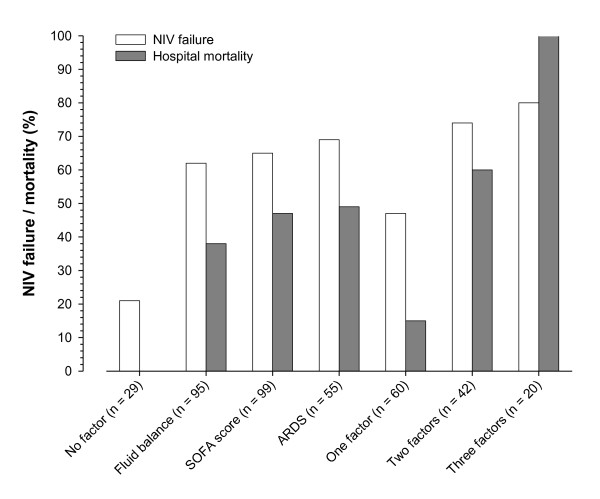
**Interaction of risk factors for failure of non-invasive ventilation and hospital mortality**. Fluid balance denotes cumulative fluid balance ≥ 2 L in the first 72 hours of intensive care unit stay. SOFA score denotes Sequential Organ Failure Assessment punctuation ≥ 4 (excluding respiratory component). ARDS denotes Acute Respiratory Distress Syndrome. *P *< 0.001 (Pearson Chi-square test) for both the comparisons of hospital mortality and non-invasive ventilation failure and risk factors interaction.

The characteristics of patients that initially received NIV are shown in Table 3. The most common diagnoses were pneumonia (23%), neurologic disorders (21%) and non-pulmonary sepsis (12%). Classical indications for NIV, such as obstructive pulmonary disease and congestive heart failure, were present in only 5% and 8% of the cases, respectively. NIV failure occurred in 54% (81/151) of patients receiving NIV initially. Factors related to NIV failure in univariate analysis were total SOFA score, SOFA score excluding respiratory component, ARDS diagnosis, length of NIV, tracheostomy, use of vasopressors and a positive cumulative fluid balance. As expected, ICU and hospital lengths of stay and mortality were higher in patients who experienced NIV failure (Table 3). In multivariate analysis, a SOFA score without the respiratory component ≥ 4 points, a diagnosis of ARDS and a cumulative fluid balance higher than 2 L in the first 72 hours of ICU stay were associated with NIV failure (Table 3). The frequency of NIV failure as well as hospital mortality increased significantly with the number of these risk factors presented by the patients (Figure 4).

**Table 3:** Factors associated with NIV failure on univariate and multivariate analysis

	Univariate comparison			Logistic regression	
	
Characteristic	NIV success (*n *= 70)	NIV failure (*n *= 81)	*P-v*alue (univariate)	OR (CI 95%)	*P-*value (multivariate)
**General**					
Age - yo	63 (48 to 80)	67 (43 to 78)	0.826		
Male gender - n (%)	34 (49)	45 (56)	0.392		
Admission SAPS 3 Score (points)	59 (50 to 68)	62 (55 to 69)	0.180		
SOFA Score on Day 1 (points)	5 (3 to 7)	7 (4 to 8)	< 0.001		
SOFA Score excluding respiratory component (points)	3 (1 to 5)	4 (4 to 7)	0.001		
SOFA excluding respiratory component ≥ 4 points	34 (48)	65 (80)	< 0.001	1.20 (1.05 to 1.34)	0.009
Charlson Comorbidity Index (points)					
0	21 (30)	25 (31)	Ref		
1 to 2	32 (46)	38 (47)	0.794		
> 2	17 (24)	18 (22)	0.782		
					
**Admission diagnoses**			0.562		
Pneumonia - n (%)	17 (24)	18 (22)			
Neurological - n (%)	13 (19)	19 (23)			
Non-pulmonary sepsis - n (%)	9 (13)	10 (12)			
Asthma/COPD - n (%)	4 (6)	3 (4)			
Cardiogenic pulmonary edema - n (%)	5 (7)	7 (9)			
Extracranial trauma - n (%)	4 (6)	2 (3)			
Hypovolemic/cardiogenic shock - n (%)	5 (7)	5 (6)			
Aspirative syndromes - n (%)	4 (7)	3 (4)			
Others - n (%)	9 (13)	14 (17)			
					
**Respiratory data**					
Inspiratory pressure first day - cmH_2_O	12 (10 to 15)	12 (11 to 15)	0.956		
Expiratory pressure first day - cmH_2_O	8 (8 to 10)	8 (6 to 10)	0.891		
PaO_2_/FiO_2 _ratio first day	230 (185 to 300)	200 (150 to 300)	0.271		
ARDS - n (%)	17 (31)	38 (69)	0.004	2.31 (1.10 to 4.82)	0.026
Length of NIV - days	3 (2 to 4)	1 (0 to 2)	< 0.001		
Day of NIV failure	---	1 (0 to 2)	---		
Tracheostomy - n (%)	2 (3)	16 (20)	0.007		
					
**ICU variables**					
Vasopressors - n (%)	16 (23)	50 (62)	< 0.001		
Cumulative fluid balance ≥ 2 L (72 h) - n (%)	36 (51)	59 (73)	0.007	2.09 (1.02 to 4.30)	0.045
					
**Outcome**					
ICU mortality - n (%)	7 (10)	38 (47)	< 0.001		
Hospital Mortality - n (%)	13 (19)	41 (51)	< 0.001		
ICU LOS - days	7 (4 to 8)	13 (7 to 23)	< 0.001		
Hospital LOS - days	14 (9 to 26)	21 (13 to 30)	0.071		

Data are expressed as median (p25 to p75) or number (%). Abbreviations: ARDS, Acute Respiratory Distress Syndrome; COPD, Chronic Obstructive Pulmonary Disease; LOS, Length-of-stay; NIV, Non-invasive ventilation; RRT, Renal Replacement Therapy; SAPS, Simplified Acute Physiology Score; SOFA, Sequential Organ Failure Assessment. Area under receiver operating curve for NIV failure (95% CI): 0.72 (0.64 to 0.80), *P *< 0.001. Hosmer-Lemeshow Χ^2^: 2.555, *P *= 0.768.

### Weaning and tracheostomy

Table 4 depicts the variables related to weaning in our population. Carrying out a spontaneous breathing trial and a successful extubation were protective factors for mortality in the univariate analysis. Additionally, 30% of the patients that were successfully extubated received non-invasive ventilation after extubation. Weaning failure with subsequent reintubation occurred in 15% of the patients. Tracheostomy was carried out in 182 (29%) patients under invasive mechanical ventilation 7 (5 to 11) days after endotracheal intubation.

**Table 4:** Weaning variables of patients under invasive mechanical ventilation

Characteristic	All patients (*n *= 622)	Survivors (*n *= 354)	Non-survivors (*n *= 268)	OR (CI 95%)	*P-*value
**General variables - n (%)**					
Successful spontaneous breathing trial	378 (61)	304 (86)	74 (28)	0.06 (0.04 to 0.09)	< 0.001
Extubation	338 (54)	270 (76)	68 (25)	0.11 (0.08 to 0.16)	< 0.001
Use of NIV after extubation	108 (17)	77 (22)	31 (12)	0.52 (0.34 to 0.83)	0.006
Reintubation	94 (15)	57 (16)	37 (14)	0.94 (0.59 to 1.47)	0.792
Time to reintubation	1 (0 to 3)	1 (0 to 3)	1 (0 to 3)	1.00 (0.94 to 1.07)	0.837
Time < 48 hours to reintubation	57 (9)	30 (8)	27 (10)	0.74 (0.38 to 1.47)	0.400
Tracheostomy	182 (29)	93 (26)	89 (33)	1.37 (0.97 to 1.39)	0.073
Days to tracheostomy	7 (5 to 11)	7 (5 to 10)	9 (6 to 12)	1.04 (0.99 to 1.10)	0.057
					
**Cause of extubation failure (more than one may apply) - n (%)**					
Coma	41 (6)	14 (4)	27 (10)	3.06 (1.57 to 5.97)	< 0.001
Agitation/delirium	11 (2)	8 (2)	3 (1)	0.54 (0.14 to 2.06)	0.371
Shock	20 (3)	3 (1)	17 (6)	8.86 (2.56 to 30.6)	< 0.001
Ventilator-associated pneumonia	13 (2)	6 (2)	7 (3)	1.73 (0.57 to 5.21)	0.330
Dyspnea	50 (8)	31 (9)	19 (7)	0.88 (0.49 to 1.61)	0.697
Cardiac dysfunction	7 (1)	2 (0.8)	5 (2)	3.71 (0.71 to 19.3)	0.118
Hypoxemia/Hypercapnia	36 (6)	16 (5)	20 (7)	1.91 (0.97 to 3.76)	0.063
Excessive respiratory secretions	24 (4)	14 (4)	10 (4)	1.05 (0.45 to 2.40)	0.912

## Discussion

In the present study, mortality rates of patients in Brazilian ICUs requiring ventilatory support were elevated, regardless of the underlying condition. Factors such as age, comorbidities, ARDS, disease severity and variables related to ICU support like positive fluid balance and NIV failure are independently associated with hospital mortality. We also observed that more than half of the patients receiving NIV as the primary modality of ventilatory support failed and required invasive mechanical ventilation subsequently. Variables independently associated with NIV failure were the severity of organ dysfunctions, the presence of ARDS and a positive fluid balance.

The mortality rate of critically ill patients under ventilatory support and patients with ARDS is elevated in both observational and interventional studies [6,21,22]. In the last decade, however, important ventilatory interventions, such as lung protective strategies with reduction in tidal volumes [3] and widespread use of NIV [23], were more frequently incorporated in the clinical practice [24] and could have resulted in different mortality rates. Nevertheless, some recent studies showed very modest or no changes in these outcomes [2,4,9,25]. In a systematic review by Phua *et al*., the pooled mortality rate of ARDS in observational studies was 48% and did not decrease significantly in the last years [26]. More recently, Villar *et al*. reported a hospital mortality rate of 48% for ARDS patients under low tidal volume ventilation [4]. We observed a higher mortality rate for the entire cohort and a more prominent rate for ARDS patients. However, our results are within the predicted mortality range of SAPS 3 and comparable to those reported in similar countries, such as Argentina [27]. Possible explanations for our findings may include unequal access to healthcare [28,29] as well as unmeasured factors related to the process of caring for these patients.

There is a significant gap between the recommendations of low tidal volumes for ARDS patients and their adoption in practice. Several observational studies demonstrated the lack of adherence to this strategy [9,30] and our study confirms these findings. Moreover, a recent meta-analysis suggests that even ventilated patients without ARDS may benefit from low tidal volumes [31]. However, it is important also to emphasize that in this trial and similar to other studies, the majority of patients were ventilated with plateau pressures below the limit of 30 cmH_2_0, which may partially compensate the harmful effects of high tidal volumes.

In our study, more than 60% of the patients under invasive MV for more than 24 hours were submitted to a spontaneous breathing trial and 54% were extubated, which is similar to previous reports [32]. Seventeen percent of the patients used NIV after extubation, an incidence also comparable to other studies [33]. Tracheostomy was done in 29% percent of the patients in a median period of one week after initiation of MV. There is significant heterogeneity in the rates of tracheostomy in patients under MV as well as at the time of the procedure [9,34-36]. A previous one-day point-prevalence study of MV that included patients from Brazil showed that tracheostomy was done in 27% of the individuals in a median of 8 (1 to 15) days after ventilatory support [12]. These numbers are similar to our report and to comparable countries such as Argentina, Chile and Uruguay [12].

NIV was used as the first line of treatment for respiratory failure in 20% of the patients in our population, with a 54% failure rate. The failure rates for NIV are quite variable in the literature and seem to be related to the cause of respiratory failure and disease severity [23,37,38]. Elevated failure rates are worrisome since NIV failure has been previously associated with increased mortality risk [39] and, in this study, was an independent risk factor for mortality. We could speculate that misperception of disease severity by the multidisciplinary team may have contributed to over-utilization of NIV for high-risk patients, delaying invasive mechanical ventilation and contributing to the poor outcome of these patients.

Another example of a potentially modifiable risk factor for mortality is related to the fluid strategy. A positive fluid balance is consistently associated with adverse outcomes in the ICU setting, mainly for patients with ARDS [40,41] and acute kidney injury [42]. We found that the extremes of cumulative fluid balance in the first three days are independently associated with hospital mortality. Interestingly, we also found that a positive fluid balance in the first days was associated with NIV failure. Our data suggest that, taking into account the hemodynamic status, a judicious fluid balance in the first days of ICU stay may be a safer goal in patients under ventilatory support.

The present study has several shortcomings. First, it was carried out during the winter period in the Southern hemisphere, and this may have influenced the incidence of respiratory infections and also the occurrence of ARDS. However, the study was conducted between early June and the end of July and epidemiologic data demonstrate that both Influenza and other lower respiratory infections present a different seasonality varying according to the region of the country [43]. We did not collect data on the origin and on the time of ARDS development, which precludes a more detailed evaluation of these patients. In addition, we used a convenience sample of ICUs that usually participate in clinical studies in Brazil and the sites that included patients are predominantly from the Southeast and Southern regions of the country, thus our data may not be representative of the entire nation. However, the concentration of hospitals from these regions is in accordance with a higher concentration of ICU beds and the healthcare system in Brazil [44]. Considering that academic institutions and those participating in clinical studies usually have better organization and standards of care, it is possible that the actual mortality of mechanically ventilated patients may be even higher.

## Conclusions

Current mortality of mechanically ventilated patients in Brazil remains elevated. Implementation of judicious fluid therapy and a watchful use and monitoring of NIV patients are potential targets to improve outcomes in this setting.

## Key messages

• Contemporary information on mechanical ventilation use in emerging countries is limited. Moreover, most epidemiological studies on ventilatory support were carried out before significant developments, such as lung protective ventilation or widespread application of non-invasive ventilation.

• In mechanically ventilated patients in Brazil, factors such as age, comorbidities, ARDS, disease severity and variables related to ICU support, such as positive fluid balance and NIV failure, are independently related to hospital mortality.

• NIV failure occurred in 54% of the patients and was associated with the severity of organ dysfunctions, presence of ARDS and positive fluid balance.

• Current mortality of ventilated patients in Brazil is exceedingly high. Implementation of judicious fluid therapy and a watchful use and monitoring of NIV patients are potential targets to improve outcomes in this setting.

## Abbreviations

AIDS: Acquired immunodeficiency syndrome; ARDS: Acute respiratory distress syndrome; BRICNet: Brazilian Research in Intensive Care Network; CI: Confidence interval; COPD: Chronic Obstructive Pulmonary Disease; ERICC: Epidemiology of Respiratory Insufficiency in Critical Care; ICU: Intensive care unit; IRB: Institutional Review Board; LOS: Length-of-stay; LOWESS: Locally weighted scatterplot smoothing; MV: Mechanical ventilation; NIV: Non-invasive mechanical ventilation; OR: Odds ratio; PEEP: Positive end-expiratory pressure; RRT: Renal replacement therapy; SAPS 3: Simplified Acute Physiology Score 3; SOFA: Sequential Organ Failure Assessment

## Competing interests

The authors declare they have no competing interests regarding the topic of this manuscript.

## Authors' contributions

All authors contributed significantly to this manuscript, including study conception (LCPA, MP, JIFS, GS, MS), data acquisition (all authors), data analysis and interpretation (LCPA, MP, MS, JIFS), drafting manuscript (LCPA, MS, JIFS), revising the manuscript for important intellectual content (all authors), and approval of the final copy (all authors).
